# Down-regulation of common NFκB-iNOS pathway by chronic Thalidomide treatment improves Hepatopulmonary Syndrome and Muscle Wasting in rats with Biliary Cirrhosis

**DOI:** 10.1038/srep39405

**Published:** 2016-12-23

**Authors:** Tzu-Hao Li, Pei-Chang Lee, Kuei-Chuan Lee, Yun-Cheng Hsieh, Chang-Youh Tsai, Ying-Ying Yang, Shiang-Fen Huang, Tung-Hu Tsai, Shie-Liang Hsieh, Ming-Chih Hou, Han-Chieh Lin, Shou-Dong Lee

**Affiliations:** 1 Division of Allergy and Immunology, Taipei Veterans General Hospital, No. 201, Sec. 2, Shipai Rd., Beitou District, Taipei City, Taiwan; 2 Department of Medicine, Taipei Veterans General Hospital, No. 201, Sec. 2, Shipai Rd., Beitou District, Taipei City, Taiwan; 3 Division of Allergy, Immunology, and Rheumatology, Department of Medicine, Chiayi Branch, Taichung Veterans General Hospital, No. 600, Sec. 2, Shixian Rd., West District, Chiayi City, Taiwan; 4 Institute of Clinical Medicine, National Yang-Ming University, Taipei, Taiwan, No. 155, Sec. 2, Linong St., Taipei, Taiwan; 5 Division of Gastroenterology & Hepatology, Taipei Veterans General Hospital, No. 201, Sec. 2, Shipai Rd., Beitou District, Taipei City, Taiwan; 6 Department of Medicine, National Yang-Ming University, No. 155, Sec.2, Linong St., Taipei, Taiwan; 7 Division of General Medicine, Taipei Veterans General Hospital, No. 201, Sec. 2, Shipai Rd., Beitou District, Taipei City, Taiwan; 8 Division of Infection Diseases, Taipei Veterans General Hospital, No. 201, Sec. 2, Shipai Rd., Beitou District, Taipei City, Taiwan; 9 Institute of Traditional Medicine, National Yang-Ming University, No. 155, Sec.2, Linong St., Taipei, Taiwan; 10 Genomics Research Center, Academia Sinica, 128 Sec. 2, Academia Rd., Nankang, Taipei City, Taiwan; 11 Cheng Hsin General Hospital, No. 45, Cheng Hsin St., Beitou District, Taipei

## Abstract

Thalidomide can modulate the TNFα-NFκB and iNOS pathway, which involve in the pathogenesis of hepatopulmonary syndrome (HPS) and muscle wasting in cirrhosis. In bile duct ligated-cirrhotic rats, the increased circulating CD16^+^ (inflammatory) monocytes and its intracellular TNFα, NFκB, monocyte chemotactic protein (MCP-1) and iNOS levels were associated with increased circulating MCP-1/soluable intercellular cell adehesion molecule-1 (sICAM-1), pulmonary TNFα/NOx, up-regulated M1 polarization, exacerbated angiogenesis and hypoxemia (increased AaPO_2_) in bronchoalveolar lavage (BAL) fluid and pulmonary homogenates. Meanwhile, a significant correlation was noted between circulating CD16^+^ monocyte/M1 (%) macrophages in BAL; M1 (%) macrophages in BAL/pulmonary iNOS mRNA expression; pulmonary iNOS mRNA expression/relative pulmonary MVD; pulmonary NOx level/AaPO_2_; circulating CD16^+^ monocyte/M1 (%) macrophages in muscle homogenates; 3-nitrotyrosine (representative of peroxynitrite) concentration/M1 (%) macrophages in muscle homogenates. The *in vitro* data demonstrated an iNOS-dependent inhibition of thalidomide on the TNFα-stimulated angiogenesis and myogenesis in human pulmonary artery endothelial cells (HPAECs) and C2C12 myoblasts. Significantly, the co-culture of CD16^+^ monocyte from different rats with HPAECs, or co-culture of supernatant of above mixed cultures with HPAECs or C2C12 myoblasts stimulated angiogenesis, migration and myogenesis. Our findings demonstrate that TNFα inhibitor thalidomide markedly diminishes the severity of experimental HPS and muscle wasting by down-regulation of common peripheral and local NFκB-iNOS pathway.

In cirrhosis, recruitment of circulating monocytes to lung tissue produces substances that trigger inducible nitric oxide synthase (iNOS)-derived nitric oxide (NO)-mediated angiogenesis and increased alveoloarterial oxygen difference (AaPO_2_) in experimental hepatopulmonary syndrome (HPS)^1^,^2^. Schenk P, *et al*. reported that cirrhotic patients with severe hypoxia (PaO_2_ < 60 mmHg) would die within 6 months^3^. Liver transplantation is the only well-established strategy to improve 5-year survival and resolution of hypoxemia for cirrhotic patients with severe HPS^4^. However, facing world-wide organ shortage crisis, it is urgent to explore potential therapeutic agents for HPS. Nuclear factor kappa B (NFκB) is an obligatory mediator of most of the tumor necrosis factor-alpha (TNFα) effects. TNFα-NFκB and iNOS-NO cascades mediate the pulmonary angiogenesis and abnormal gas exchange in experimental HPS of biliary cirrhosis^2^,^5^,^6^. TNFα neutralization has been reported to alleviate the cirrhotic HPS through the inhibition of iNOS-NO pathway^6^.

Muscle wasting is a frequent cirrhotic complication that contributing to increased sepsis-related and post-transplantation mortalities^7^,^8^. Gayan-Ramirez G, *et al*. firstly explored the pathogenesis of skeletal muscle wasting in experimental biliary cirrhosis^9^. In biliary cirrhosis, the up-regulated muscle TNFα pathway had been reported to be resulted in protein degradation and muscle wasting^10^,^11^. Administration of TNFα receptor antagonist had inhibited TNFα-related muscle wasting cascades in rats with biliary cirrhosis^12^. However, the pathogenic roles of recruited muscular macrophages and activated TNFα-NFκB-iNOS cascades have not yet been explored in muscle wasting of cirrhosis.

Cirrhosis is a chronic inflammatory syndrome that characterized by persistent activation of monocyte/macrophage and high plasma TNFα/monocyte chemoattractant protein-1 (MCP-1) levels^13^,^14^. Significant expansion of circulating CD16^+^ (inflammatory) monocytes has been reported in cirrhotic patients^15^. MCP-1 has been reported as the main chemokine to recruit CD16^+^ monocytes, which express tissue TNFα and iNOS in liver fibrosis model^16^. TNFα, MCP-1 and iNOS-derived NO are pro-inflammatory M1 cytokines that responsible for M1 macrophages polarization^17^. In chronic inflammatory state, M1 macrophages polarization is involved in muscle wasting^18^. In cirrhosis, recruitment of circulating leukocyte to lung and peritoneal cavity is a chemoattractant-dependent process that involves the activation of TNFα-NFκB-iNOS cascades^5^,^19^,^20^. In tissue with chronic inflammation, increased inflammatory monocytes and M1 macrophages polarization result in angiogenesis and protein degradation^6^,^18^,^21^,^22^.

Infiltrated macrophages enhanced iNOS-derived NO production and depletion of macrophages was reported to prevent and regress HPS^21^. In muscular dystrophy, M1 macrophages result in severe muscle wasting via iNOS-dependent mechanism^22^. Transcription factor myogenic differentiation gene (MyoD) regulates skeletal muscle differentiation/maintenance and is essential for repair of damaged tissue^23^. TNFα, a mediator of skeletal muscle wasting, stimulates NFκB-dependent MyoD down-regulation and skeletal myofibers dysfunction^24^. Actually, NFκB-mediated MyoD decay during muscle wasting is also an iNOS-dependent process^25^.

Thalidomide attracts us as a potential treatment for HPS and muscle wasting due to previous well-reported beneficial effects including amelioration of hepatic fibrosis, portal hypertension, hyperdynamic circulation and mesenteric angiogenesis in experimental cirrhotic models^26^,^27^,^28^. Meanwhile, thalidomide is a TNFα inhibitor that down-regulating the NFκB-iNOS pathway^29^, which simultaneously contributes to HPS and muscle wasting in cirrhosis. Nonetheless, the potential effects and mechanisms of chronic thalidomide on the HPS and muscle wasting have never been explored in cirrhosis.

In cirrhosis, effective medical therapies for HPS and muscle wasting have yet to be established. In this study, we evaluated the effects of the modulation of common NFκB-iNOS pathway by chronic anti-TNFα treatment with thalidomide on the HPS and muscle atrophy of cirrhotic rats. Thoughtfully, the *in vivo* and *in vitro* mechanisms and effects of chronic thalidomide on the angiogenic and atrophic pathways in rat cirrhotic lung and skeletal muscle were elucidated.

## Results

### Systemic effects of chronic thalidomide treatment

In comparison with S-V rats, significantly higher plasma TNF-α, MCP-1, sICAM-1, vascular endothelial growth factor (VEGF), ALT and AST levels and hepatic hydroxyproline as well as lower MAP were noted in the Bile-duct ligation-vehicle (BDL-V) rats (Table 1), which were significantly reversed by current dose and duration of thalidomide treatment in Bile-duct ligation-thalidomide (BDL-thal) rats (Table 1).

### Thalidomide treatment decreased circulating CD16^+^ (inflammatory) monocyte subsets proportion

**Figure 1:**
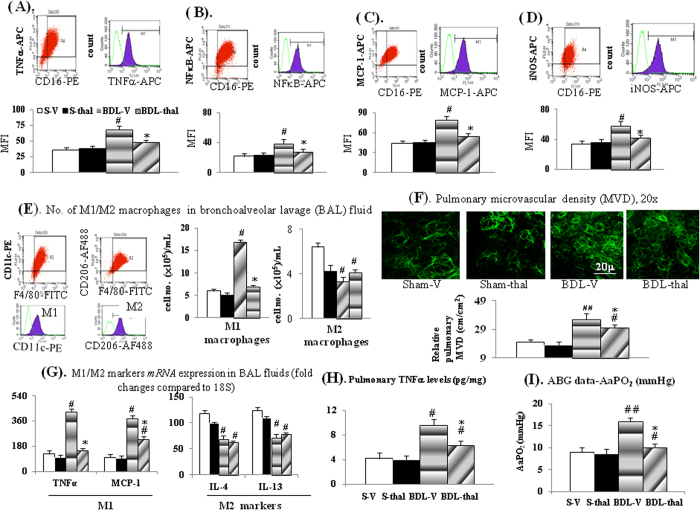
Effects of thalidomide on rat circulating monocyte and pulmonary macrophages. (**A–D**) Intracellular cytokines levels in circulating CD16^+^ monocytes; (**E**) number of M1/M2 macrophages [F4/80(+)/CD11c(+)cells]/[F4/80(+)/CD206(+) cells] and M1/M2 genes expression (**G**) in BAL fluid; (**F**) pulmonary relative MVD (microvascular density); (**H**) pulmonary TNFα levels; (**I**) Arterial blood gas (ABG) data-A-a gradient (AaPO_2_); ^#,##^*p* < 0.05, 0.01 *vs*. S-V; *p < 0.05 *vs*. BDL-V.

Percentage (%) of circulating CD16^+^ monocyte in total monocytes and its intracellular levels of TNF-α, NFκB, MCP-1 and iNOS were significantly higher in BDL-V rats than those in S-V rats, and chronic thalidomide treatment inhibited the elevated circulating CD16^+^ monocytes in BDL-thal rats (Table 1, Fig. 1A–D).

### *In vivo* effects of thalidomide treatment on cirrhotic lung

**Figure 2:**
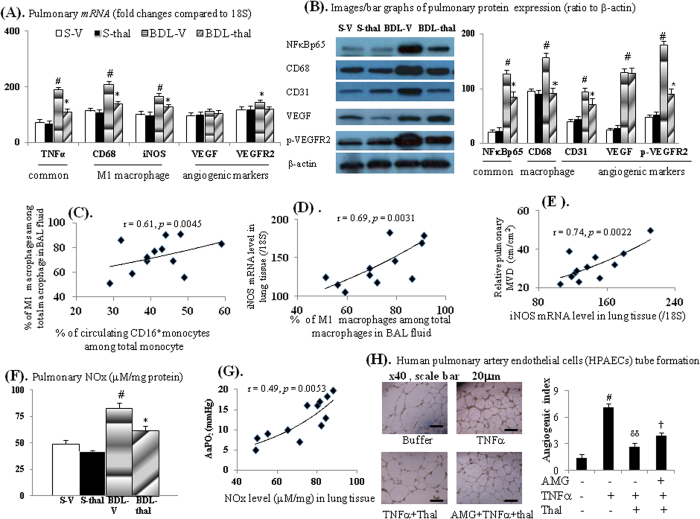
Effects of thalidomide on cirrhotic lungs. (**A**,**B**) *mRNA/*protein expressions. (**C**) correlation between M1 macrophages (%) in BAL fluid and circulating CD16^+^ monocyte (%); (**D**) correlation between M1 macrophages (%) in BAL fluid and iNOS *mRNA* level in lung; (**E**) correlation between iNOS *mRNA* level in lung and relative pulmonary MVD; (**F**) total pulmonary nitric oxide (NOx) level; **(G)** correlation between pulmonary NOx level and AaPO_2_ (mmHg); (**H**) HPAECs tube formation after various treatments. ^#,##^*p* < 0.05, 0.01 *vs*. S-V (buffer only group); *p < 0.05 *vs*. BDL-V; ^δ^*p* < 0.05 *vs*. TNFα groups; ^†^*p* < 0.05 *vs*. TNFα + thalidomide (thal) groups.

Up-regulated TNFα/NFκBp65 expression, higher M1 macrophages number and M1 (TNFα, MCP-1, CD68 and iNOS) markers expression, lower number of M2 macrophage and M2 (IL4 and IL-13) markers expression in BAL fluid and lung tissue were associated with higher pulmonary TNFα levels in BDL-V rats compared to those of S-V rats (Figs 1E,G,H and 2A,B). Significantly, two-week thalidomide treatment reversed above-mentioned abnormalities in BDL-V rats except for M2 macrophages number and M2 *mRNA*s [interleukin-4 (IL-4) and interleukin-13 (IL-13)] expression in BAL fluid of BDL-thal rats (Fig. 1E,G). In comparison with S-V rats, higher pulmonary vascular density (MVD) and angiogenic (iNOS, VEGF and p-VEGFR2) marker expressions were noted in the BDL-V rats (Figs 1F and 2A,B), which were effectively diminished by thalidomide treatment in BDL-thal rats.

Notably, the percentage of circulating CD16^+^ monocyte was positively correlated with the percentage of BAL fluid M1 macrophage in BDL-cirrhotic rats (Fig. 2C). BAL fluid M1 macrophage was positively correlated with the pulmonary iNOS *mRNA* level, which also was positively correlated with relative pulmonary MVD in BDL-cirrhotic rats (Fig. 2C–E). In BDL-V rats, the increased pulmonary NOx levels and above-mentioned pulmonary angiogenic markers were associated with increased A-a gradient (AaPO_2_) (Figs 1I and 2F). A positive correlation was also noted between pulmonary NOx level and AaPO_2_ of BDL-cirrhotic rats (Fig. 2G). Effectively, thalidomide treatment decreased pulmonary NOx levels/angiogenic markers and A-a gradient (AaPO_2_) of BDL-thal rats.

Notably, up-regulated *mRNAs* expression of pulmonary endothelin-1 (ET-1), endothelin B receptor (ET_B_R) and eNOS were noted in BDL-V rat lung than those in S-V lung tissues [Supplement Table 2]. However, the *mRNA* expressions of pulmonary ET-1, ET_B_R and eNOS were not different between BDL-V and BDL-thal rat lungs as well as between S-V and S-thal rat lung tissues [Supplement Table 2].

### Direct *in vitro* effects of thalidomide on human pulmonary artery endothelial cells (HPAECs)

**Figure 3:**
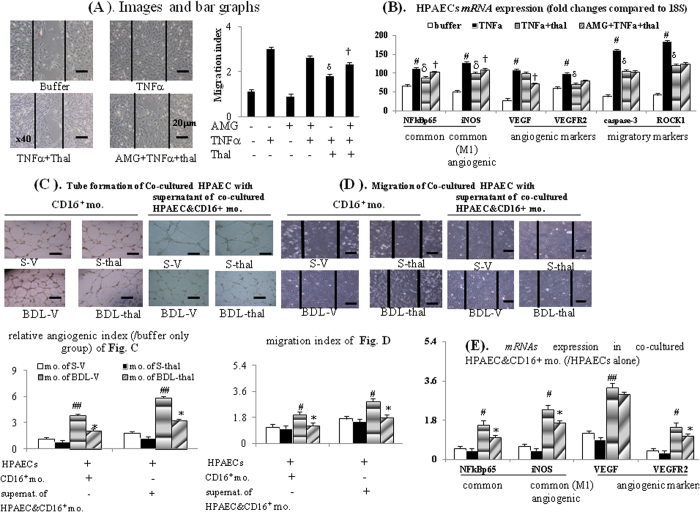
*In vitro* effects of thalidomide on HPAECs. (**A**) HPAECs migration assays after various treatments; (**B**) various *mRNA* expression in HPAECs’ cell lysates; (**C**) angiogenic, (**D**) migration index in co-cultivation of CD16^+^ monocyte from different rats with HPAECs or system of co-incubated HPAECs with supernatant of co-cultured CD16^+^ monocyte + HPAECs; (**E**) ratio of *mRNA* expressions in co-cultured CD16^+^ monocyte+HPAECs to those in mono-cultured HPAECs. ^#,##^*p* < 0.05, 0.01 *vs*. S-V; *p < 0.05 *vs*. BDL-V; ^δ^*p* < 0.05 *vs*. TNFα groups; ^†^*p* < 0.05 *vs*. TNFα+thalidomide (thal) groups.

As presented in Figs 2H and 3A,B, TNFα incubation stimulated the HPAECs tube formation and migration as well as common (NFκBp65/iNOS), angiogenic (iNOS/VEGFR2), migratory (caspase-3/ROCK1) factors *mRNA* expressions, which both were significantly inhibited by acute thalidomide co-incubation. Meanwhile, 2-hour pre-treatment with AMG (aminoguanidine, an iNOS inhibitor) significantly reduced the suppressive effects of thalidomide on above mentioned TNFα-stimulated common/angiogenic/migratory cascades (2H & 3A-B).

In Fig. 3A, the increased migration index (MI) in TNFα group than buffer only group was not totally abolished by AMG 2-hour pre-treatment in AMG + TNFα groups. Meanwhile, AMG pre-treatment in AMG + TNFα + thal group did not completely reversed the suppressive effects of thalidomide on TNFα-stimulated HPAEC migration compared to TNFα + thal and TNFα group (Fig. 3A). These results suggested that TNFα-stimulated HPAEC migration was mediated both by iNOS and other factors (caspase-3/ROCK1).

Co-cultured with CD16^+^ monocytes collected from BDL-V rats, HPAECs tube formation and migration were significantly more than those co-cultured with CD16^+^ monocytes from S-V rats; furthermore HPAECs tube formation and migration were suppressed in those co-cultured with CD16^+^ monocytes from BDL-thal rats (Fig. 3C,D). Then, the supernatant of co-cultured systems of HPAECs and CD16^+^ monocytes collected from different groups were co-cultured with HPAECs (Fig. 3C,D). Separately, S-V, BDL-V, S-thal, BDL-thal group indicated co-cultured supernatant of co-cultured systems of HPAECs and CD16^+^ monocytes collected from S-V, BDL-V, S-thal, BDL-thal rats. Noteworthy, the HPAECs migration and tube formation in the BDL-V group significantly augmented, and these effects likewise suppressed in that of co-cultured with supernatant of BDL-thal group (Fig. 3C,D). The *mRNA* expressions of NFκBp65/iNOS (common/M1) and VEGF and VEGFR2 (angiogenic), were significantly up-regulated in co-cultured HPAECs and CD16^+^ monocytes collected from BDL-V rats, compared to which were suppressed in those from BDL-thal rats (Fig. 3E). In other words, the *in vitro* HPAECs tube formation and migration could be induced directly by CD16^+^ monocytes and indirectly by releasing M1 and angiogenic factors in supernatant of co-cultured HPAECs and CD16^+^ monocytes.

### *In vivo* effects of thalidomide treatment on cirrhotic muscle

**Figure 4:**
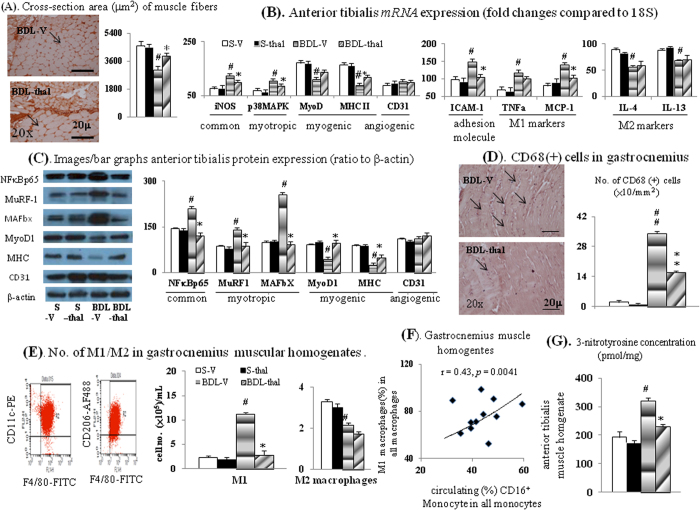
Effect of thalidomide treatment on the cirrhotic muscles. IHC images for measurement of (**A**) cross-section area of muscle fibers with α-sarcometric actin antibody and (**D**) macrophages infiltration with CD68 antibody; (**B,C**) various *mRNA*s/proteins expression; (**E**) numbers of M1/M2 macrophages in muscle homogenates; (**F**) correlation between muscular M1 macrophages (%) in BAL fluid and circulating CD16^+^ monocyte (%); (**G**) 3-nitrotyrosin (representative of peroxynitrite) concentration in anterior tibialis muscle homogenates. ^#,##^*p* < 0.05, 0.01 *vs*. S-V; *p < 0.05 *vs*. BDL-V.

Table 2 revealed that thalidomide treatment significantly normalized the absolute/relative skeletal muscle weight, cross-section area of muscle fiber and muscular protein contents in BDL-V rats, which were significant less than S-V rats (Fig. 4A and Table 2). In comparison with S-V rat muscles, the increased expressions of common (iNOS/NFκBp65), myotripic [p38MAPK/muscle RING-finger protein family 1 (MuRF1)/muscle atrophy F-box (MAFbx)] and decreased myogenic [MyoD1/myosin heavy chain (MHC-II)] markers were noted in BDL-V rat muscles (Fig. 4B,C), and these abnormalities were corrected in BDL-thal rat muscles. Notably, the normal muscular angiogenesis in BDL-V rat was not compromised by thalidomide treatment in BDL-thal rat muscles (not different in CD31 expression).

Compared with S-V rat muscles, adhesion molecule (ICAM-1), M1 (TNFα and MCP-1) markers expressions and infiltrated M1 macrophage (CD68 IHC staining and muscle homogenates) were significantly increased in BDL-V rat muscles, and these abnormalities were corrected in BDL-thal rat muscles (Fig. 4B,D,E).

**Figure 5:**
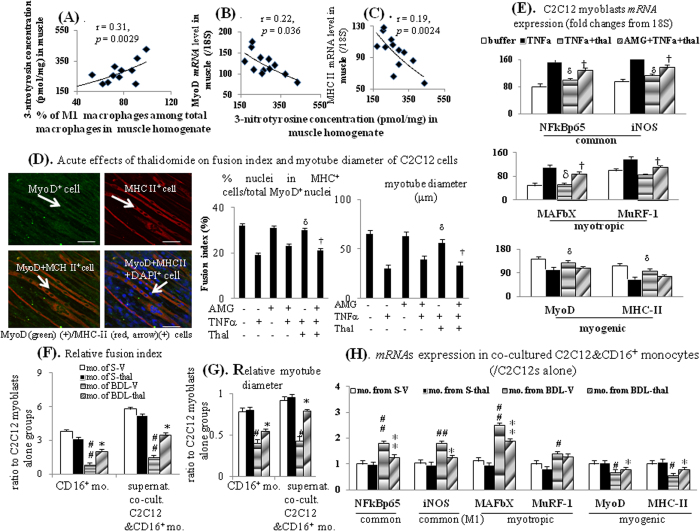
Effects of thalidomide treatment on myogenic profiles. **(A)** Correlation between muscular 3-nitrotyrosine concentration and M1 macrophages (%); correlation between muscular (**B**) MyoD and (**C**) MHC II *mRNA* expression with 3-nitrotyrosine concentration in anterior tibialis muscle homogenates. (**D**) Representative immunofluorensence (IF) image and bar graphs of acute effects of thalidomide on fusion index and myotube diameter of C2C12 cells; (**E**) various *mRNA* expression in C2C12s’s cell lysates; **(F)** relative fusion index and **(G)** myotube diameter in co-culture of CD16^+^ monocyte collected from different rats with C2C12 cells or co-incubation of supernatant of co-cultured CD16^+^ monocyte + C2C12 cells with C2C12 cells; (**H**) ratio of *mRNA* expressions in co-cultured CD16^+^ monocyte+C2C12 cells to those in mono-cultured C2C12 cells. ^#,##^*p* < 0.05, 0.01 *vs*. S-V; ^*,**^p < 0.05, 0.01 *vs*. BDL-V; ^δ^*p* < 0.05, 0.01 *vs*. TNFα groups; ^†^*p* < 0.05 *vs*. TNFα+thalidomide (thal) groups.

In addition, a positive correlation was noted between the percentage of muscular M1 macrophage and proportion of circulating CD16^+^ monocytes in BDL-cirrhotic rats (Fig. 4F). Significantly, increased muscular 3-nitrotyrosine (marker of peroxynitrite) level was observed in BDL-V rats than S-V rats, and thalidomide treatment that in BDL-thal rats (Fig. 4G). In BDL-cirrhotic rat muscles, the 3-nitrotyrosine level was positively related to infiltrated M1 macrophage percentage (Fig. 5A) and negatively related to MyoD and MHC II *mRNA* expressions (Fig. 5B,C).

### Direct *in vitro* effects of thalidomide on C2C12 myoblasts

As presented in Fig. 5D,E, TNFα pre-incubation up-regulated NFκBp65/iNOS and myotropic (MAFbX and MuRF-1) markers *mRNA* expressions, down-regulated myogenic markers (MyoD and MHC II) expressions and inhibited the C2C12 myoblasts fusion and tube formation, which both were significantly improved by concomitant acute thalidomide incubation; notably, these effects produced by thalidomide were reduced by AMG (pre-treatment 2-hour before TNFα or TNFα + thal stimulation).

Notably, the trend of increased in fusion index/myotube diameter of C2C12s in AMG+TNFα group compared to TNFα group were noted. In AMG+TNFα+thal group, AMG pre-treatment almost totally reversed the restorative effects of thalidomide on TNFα-suppressed fusion index/myotube diameter observed in TNFα + thal group. In other words, the fusion index/myotube diameter in AMG+TNFα + thal group was almost similar to those in TNFα group (Fig. 5D). These results suggested that TNFα-suppressed C2C12 fusion index/myotube diameter, which could be reversed by acute thalidomide co-incubation, was mainly mediated by iNOS.

Co-cultured with CD16^+^ monocytes from BDL-V group, C2C12 myoblasts fusion and myotube formation were significantly less than those co-cultured with CD16^+^ monocytes from S-V group; furthermore these suppressive effects were corrected in those co-cultured with CD16^+^ monocytes from BDL-thal group (Fig. 5F,G). Co-cultured with the supernatant from the previous co-cultivation of C2C12 myoblasts and CD16^+^ monocytes in each group, C2C12 myoblasts fusion and myotube formation in the BDL-V group significantly reduced, and these effects likewise corrected in that of co-cultured with supernatant of BDL-thal group (Fig. 5F,G). The NFκBp65/iNOS and myotropic markers expressions were significantly up-regulated, whereas *mRNA* expressions of myogenic markers were down-regulated in system of co-cultured C2C12 myoblasts with CD16^+^ monocytes from BDL-V group, compared to which these effects were corrected in those from BDL-thal group (Fig. 5H).

## Discussion

With thalidomide treatment in cirrhotic rats with HPS, we found that up-regulated pulmonary TNFα-NFκB and iNOS pathways were normalized and pulmonary angiogenesis and hypoxemia were improved. Additionally, our study characterized by using organ specific cell line HPAECs and discovered that thalidomide effectively suppressed *in vitro* capillary tube formation, transwell migration and pathogenic genes expression. Previous studies had reported that the TNFα can induce apoptosis protein (caspase) release that following by caspase-mediated Rho-associated protein kinase (ROCK) activation^30^,^31^,^32^. Two isoforms of ROCK including ROCK1 and ROCK2 with highly homologous have been described^33^. Increased expression of ROCK1 in endothelial cell migration pathways can cause an increase in angiogenisis^34^,^35^. There is a reciprocal positive regulating loop between apoptosis protein and ROCK expression^33^. In addition to suppression of TNFα-induced angiogenic factors, our study suggested that thalidomide inhibited the TNFα-induced migratory factors release in cultured HPAECs.

**Figure 6:**
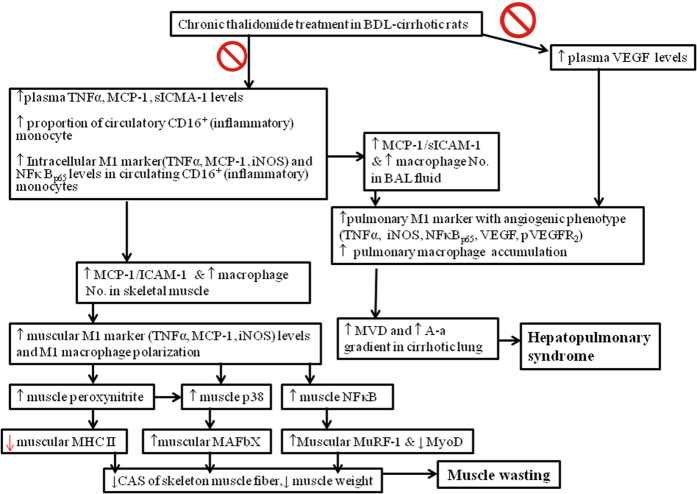
Schematic representative hypothesis for anti-sarcopenia and anti-angiogenesis effect of chronic thalidomide treatment on cirrhotic rats of our study. Abbreviations: BDL: bile duct ligation; TNFα: tumor necrosis factor-alpha; MCP-1: monocyte chemoattractic protein-1; VEGF: vascular endothelial growth factor; iNOS: inducible nitric oxide synthase; NFκB: nuclear factor kappa B; HPAEC: human pulmonary artery endothelial cells; BAL: bronchoalveolar lavage; MVD: microvascular density; MHC: myosin heavy chain.

Recently, the pathogenic roles of TNFα-NFκB and iNOS pathways have been widely explored and confirmed in non-cirrhotic muscle wasting models^36^,^37^,^38^. Different from the well-established roles of TNFα-NFκB and iNOS pathways in the pathogenesis of severe HPS in cirrhosis, only role of TNFα rather than whole TNFα-NFκB and iNOS cascades has been explored in experimental cirrhotic models with muscle wasting^10^,^11^,^12^. Our study was characterized by clarification of the mechanisms of NFκB-iNOS-mediated effects of thalidomide on muscle wasting in rats with biliary cirrhosis (Fig. 6).

Loss of myogenic MyoD is known to prevent the differentiation of myoblasts to myotubes and subsequent myofilaments/muscle fibers^36^. The over-expression of myotropic MAFbx and MuRF1 can lead to severe protein degradation and muscle atrophy^37^. Our *in vitro* study with C2C12 myoblasts revealed that the acute TNFα incubation could up-regulated myotropic MAFbx/MuRF1 and down-regulated myogenic MyoD expression. A key mechanism of NFκB-mediated muscle atrophy involves the activation of the protein degradation pathway by the down-regulation of MyoD and up-regulation of the MAFbx/MuRF1^23^,^24^,^36^. Further study revealed that increased muscular iNOS expression is an additional mechanism for NFκB-mediated muscle wasting^38^. Our *in vitro* studies revealed that the normalization of MyoD, MAFbx and MuRF1 expression in cultured C2C12 myoblasts with acute thalidomide was mediated by iNOS. Under pathogenic conditions, iNOS-derived NO reacts with superoxide anions (O_2_^−^) to form the toxic molecule peroxynitrite (ONOO^−^), which will result in MyoD *mRNA* decay and muscle atrophy^33^. Additionally, recent study suggested the increased muscular nitration play important roles in the pathogenesis of muscle wasting in rats with biliary cirrhosis^11^. In our cirrhotic rat skeletal muscle, the decreased protein content, down-regulated myogenic MyoD expression and up-regulated myotropic iNOS/MAFbx/MuRF1 expression were normalized by chronic thalidomide treatment with blocking of TNFα-NFκB cascades. Consequently, the decreased relative wet weight of hindlimb muscle and diminished cross-section area of skeletal muscle fiber were reversed by chronic thalidomide treatment in our cirrhotic rats.

Furthermore, the NFκB-iNOS-mediated MyoD loss has been correlated with MHC loss in myotubes that lead to muscle wasting^36^,^37^. It is noteworthy that peroxynitrite can induce degradation of muscle proteins by activation of p38 MAPK and up-regulation of the myotropic MAFbx and MuRF1^39^,^40^. In paralleled to decreased MyoD and MHC expression, the increased 3-ntrotyrosin (representative of peroxynitrite content) was accompanied by up-regulated p38 MAPK/MAFbx/MuRF1 expression in our cirrhotic muscle, which was normalized by chronic thalidomide treatment.

Collectively, our studies support the pathogenic roles of iNOS-derived NO in the pathogenesis of cirrhotic muscle wasting like previously documented in non-cirrhotic muscle wasting^36^,^37^. Thus, given the shared involvement of iNOS in mediating NFκB-mediated HPS and muscle wasting, any therapy that targets the iNOS-NO pathway may prove to be an effective therapeutic strategy in both diseased states^2^,^6^,^11^. Simultaneously, blocking TNFα-NFκB and iNOS-NO cascades with thalidomide ameliorated both HPS and muscle wasting in our cirrhotic rats.

The increased circulating CD16^+^ monocytes and its intracellular TNFα/NFκB/iNOS/MCP-1 levels, circulating chemokines (MCP-1)/adhesion molecules (sICAM-1) levels were associated with the pulmonary macrophages recruitment in our rat cirrhotic rats with experimental HPS. Remarkably, our experimental results in addition to previous observations provide more complete pathogenic mechanisms for the development of cirrhotic HPS^2^,^6^,^21^. Promisingly, chronic thalidomide treatment effectively reversed abovementioned abnormalities and ameliorated HPS in our cirrhotic rats.

Our study strengthens the concepts of systemic increased MCP-1 and ICAM-1 attract circulating monocyte to tissues including cirrhotic lung and muscle^5^,^16^,^19^,^20^. In obese mice, chronic thalidomide treatment was reported to decrease MCP-1 expression and macrophages infiltration in adipose tissue^41^. It has been reported that high serum ICAM-1 level is associated with high mortality in cirrhotic patients^42^. Further study observed that chronic thalidomide treatment inhibited the hepatic adhesion molecules expression in cirrhosis^43^. Likewise, chronic thalidomide treatment significantly diminished MCP-1 and ICAM-1 expression in circulation, BAL and muscle homogenate of our cirrhotic rats. Overall, current study revealed a significant association between abovementioned circulating dysregulation and increased macrophages infiltration in cirrhotic muscle. Subsequently, infiltrated macrophages stimulate local NFκB-iNOS cascades and downstream muscle wasting signals in our cirrhotic rats. Parallelly, these *in vitro* studies by means of CD16^+^ monocytes and HPAECs/C2C12 myoblasts co-culture system support the *in vivo* observations that increased circulating CD16^+^ monocytes is a common initiators for the development of severe HPS and muscle wasting in our BDL-cirrhotic rats.

In current study, *in vitro* co-cultured experiments were carefully studied to avoid the loss of polarity of CD16^+^ monocytes when broken away from the pathological stimulation *in vivo* for long period. Notably, the flow cytometry-measured intracellular M1 factors (TNFα and iNOS) levels were significantly higher in primary CD16^+^ monocytes collected from BDL-V rats than those in CD16^+^ monocytes collected from S-V rats in current study (Fig. 1A,D). This result indicated that the polarity of primary CD16^+^ monocytes had been preserved which supported by consistently increased expression of TNF-α and iNOS. Along with preserved polarity of primary CD16^+^ monocytes, higher angiogenic/migration/fusion index were noted in HPAEC/C2CA12 that co-cultured with CD16^+^ monocytes collected from BDL-V rats than HPAEC/C2CA12 that co-cultured with CD16^+^ monocytes collected from S-V rats (Figs 3C,D and 5F,G). Meanwhile, the levels of M1 factor (iNOS) in supernatant of co-cultured HPAEC&CD16^+^ monocytes were significant higher in BDL-V group compared to S-V group. Recently, it had been reported that M1 factors (TNFα) can directly increase VEGF-induced angiogenic/migration index of HUVECs^28^. Accordingly, it is possible that HPAECs tube formation/migration or C2CA12 myotube formation were induced either directly by CD16^+^ monocytes or indirectly by releasing M1 factors in current study.

Thalidomide treatment inhibits pulmonary and muscular M1 macrophages polarization and cytokines release in addition to suppressing percentage of circulating CD16^+^ monocytes^44^,^45^,^46^. Furthermore, unremitting inflammatory response-activated M1 macrophages induce muscle atrophy through the production of cytotoxic levels of NO by iNOS^38^. Significantly, our current studies revealed that the inhibition of M1 macrophage polarization by chronic thalidomide treatment decreased cirrhotic pulmonary and muscular iNOS-NO activity and peroxynitrite levels. Collectively, the common pathogenic mechanism in circulation is increased proportion of CD16^+^ (inflammatory) monocytes whereas in tissue is skew balance to M1 macrophage for HPS and muscle wasting in cirrhosis. Correspondingly, the levels of main regulatory cytokines in circulating inflammatory cytokines, plasma, BAL fluid and muscle homogenates were also effectively inhibited by chronic thalidomide treatment in our biliary cirrhotic rats with HPS and muscle wasting (Fig. 6).

It had been documented that ET-1-ET_B_R-eNOS pathway can interact with TNFα cascades to trigger pulmonary microvascular changes of experimental HPS in biliary cirrhotic rats^47^,^48^. Previous studies suggested that the expressions of eNOS and ET-1 could be modulated (both stimulation and inhibition) by TNFα^49^,^50^,^51^. In the pathogenesis of cirrhotic HPS, the activated eNOS-NO cascades were mainly stimulated by ET-1-ET_B_R signals^47^,^48^. In current study, the up-regulated pulmonary ET-1, ET_B_R and eNOS mRNA expressions in BDL-V rat lung tissues were not modified in BDL-thal rat lung tissues. In other words, blocking of TNFα by chronic thalidomide treatment could not suppress the up-regulated pulmonary ET-1-ET_B_R-eNOS signals in our current study of experimental cirrhotic HPS. Accordingly, chronic thalidomide treatment should be combined with other agents such as selective ET_B_ receptor antagonist to prevent recurrence of cirrhotic HPS after single usage of thalidomide in clinical practice.

In experimental cirrhotic studies, the therapeutic effects of specific agent will be more informative after verification of their beneficial effects in another cirrhotic model [carbon tetrachloride (CCL4) or Thioacetamide (TAA) for example)]. However, the biliary (BDL) cirrhosis is the only well-documented models for cirrhotic HPS^2^,^5^,^6^,^21^ and the most studied model for cirrhotic muscle wasting^9^,^10^,^11^,^12^. In other words, BDL cirrhotic model is the most suitable experimental model for simultaneous assessment of cirrhotic HPS and muscle wasting.

In conclusion, combination with previous beneficial effects of thalidomide on peripheral, hepatic and splanchnic circulations, the current studies support that future clinical trial of thalidomide may be reasonable in cirrhotic patients.

## Materials and Methods

The detail description was shown in Supplement Materials and Methods.

### Dose of thalidomide administration

Cirrhosis was induced in adult male Sprague-Dawley rats (250–350 g) by bile-duct-ligation (BDL)^27^,^28^. This study was approved by the Animal Experiment Committee of Yang-Ming University and conducted according to the “Guides for the care and use of laboratory animals” prepared by the National Academy of Science, USA.

Two weeks of thalidomide (50 mg/kg, 100 mg/kg, 200 mg/kg/day) by oral gavages was given to BDL rats in a dose-finding study (n = 3 each dose). Interestingly, the most potent suppression of TNFα *mRNA* levels in homogenates of lung and gastrocnemius was presented at 100 mg/kg/day of thalidomide. Then, experiments were performed on BDL-cirrhotic rats that had randomly received 2-week vehicle (distilled water, C-V) or thalidomide from the 2-weeks after BDL (BDL-thal) (n = 7 in each group). In other words, all the experiments were performed 1-month after BDL or sham operation of all rats.

### Experimental design

In the first set of animals (n = 7 in each group), the following experiments were performed sequentially after trachea cannulation using PE-240 polyethylene tube under anesthesia. After body weight, mean arterial pressure (MAP), heart rate (HR), and arterial blood gas (ABG) measurements, heparinized whole body blood was collected (25–30 mL from each rat) for peripheral blood mononuclear cells (PBMC) isolation [yield ≈ 30–35 × 10^6^ PBMC] for subsequent CD16^−^/CD16^+^ monocyte fraction separation and flow cytometry-based assessment of intracellular cytokines levels on CD16^+^ monocytes. Immediately after scarification, bronchoalveolar lavage (BAL) was collected from pre-cannulated trachea tube for pulmonary M1/M2 phenotype macrophages numbers measurement, pulmonary angiogenesis immunofluorescence (IF) study was performed on fresh right lung, right gastrocnemius homogenates were used for isolation and calculation of muscle M1/M2 macrophages, pulmonary TNFα/NOx levels and various protein/mRNAs were measured in left lung homogenates, immunohistochemical (IHC)-assessed macrophage infiltration and cross-section area of muscle fiber were measured in paraffin-embedded left gastrocnemius tissue section, protein content/3-nitrotyrosine concentration, and *mRNA*/protein expressions were measured in bilateral anterior tibilais homogenates and hepatic hydroxyproline levels were evaluated in liver homogenates.

In the second set of animals, the heparinized whole body blood (25–30 mL from each rat) collected from inferior vena cava, abdominal aorta, and heart chamber under anesthesia was divided into two parts. The first part, 10 mL of heparinized blood collected from each rat, was used for various serologic markers measurements. For second part, 15 mL of heparinized blood from each rat was pooled [total 75 mL of whole blood from each group of BDL-V, BDL-thal, C-V, C-thal rats (n = 7 in each group)] to prepare CD16^+^ monocyte fractions for co-culture with either HPAECs or C2C12 myoblasts. For assessment of acute effects of thalidomide on the TNFα-modulated cascades, in *vitro studies* were undergone with HPAECs and C2C12 myoblasts.

### Systemic haemodynamics and ABG analysis

The right femoral artery was cannulated for MAP and HR measurement. Femoral arterial blood (0.25 mL) at rest was used for ABG analysis using ABL 520 radiometer (radiometer America, Westlake, OH, USA) and the alveolar-arterial oxygen gradient was calculated as 150-(Pa_CO2_/0.8)-Pa_O2_. Where Pa_CO2_ is the partial pressure of carbon dioxide and Pa_O2_ is the partial pressure of oxygen. By mid-line abdominal incision, cirrhosis was confirmed by hard, nodular liver and splenomegaly.

### Proportion of CD16^+^ (inflammatory) monocyte subsets between groups

Carefully, all the heparinized blood (25–30 mL) in the inferior vena cava, abdominal aorta, and heart chamber were collected. After centrifugation, PBMCs (25–30 × 10^6^) were isolated from buffy coats of each rat by density-gradient centrifugation using endotoxin-free Ficoll-Hypaque (GE-Healthcare). To obtain the CD14^+^ monocyte fraction, CD56^−^CD16^−^ PBMCs were incubated with MACS anti-CD14 FITC-conjugated antibody. Immediately, CD56^−^CD16^−^CD14^+^ and CD56^−^CD16^+^ CD14^+^ cells (referred to as CD16^−^ and CD16^+^ monocytes) were separated by magnetic cell sorting, using MACS isolation kits by negative selection^52^. Then, the proportion of circulating CD16^−^CD14^+^ and CD16^+^CD14^+^ monocytes between groups was calculated and % of CD16^+^ monocytes among total monocytes were compared between groups.

### Intracellular cytokines assays on CD16^+^ monocytes

Isolated CD16^+^ monocytes were plated at a density of 1 × 10^5^ cells per well in a 96-well dish. For intracellular inflammatory/regulatory cytokine analysis, the cells were double-stained with anti-CD16-PE and anti-TNFα-APC, anti-NFκBp65-APC, anti-MCP-1 (monocyte chemotactic protein)-APC, anti-iNOS-APC, or isotype-matched IgG monocloned antibodies for 1-hour. After washing in 1% fetal bovine serum (FBS) in PBS, the cell were re-suspended in 2% formaldehyde for 30 minutes and washed again. The cells were re-suspended in the solution consisting of 10% dimethyl sulfoxide (DMSO) and 90% FBS. Then, all cells were thawed and washed in 1% FBS, and various intracellular cytokine levels were analyzed by flow cytometry (FACScan, BD Biosciences). For flow cytometry, the MFI of the isotype control was subtracted from the MFI of antibody-stained cells for each culture. The MFI data of rats of different groups in three independent measurements were analyzed using FlowJo software (Tree Star, Ashland, OR) and compared between groups.

### BAL fluid collection

To obtain BAL fluid from each sacrificed rats to analyze the portion (%) of M1 macrophages among total macrophages, the right lung was washed gently with 1 mL of sterile 1% FCS in phosphate-buffered saline (PBS) through cannulated trachea for six times. The fluid recovered after each aliquot instillation was combined into one FACS tube (approximately 4–5 mL) and centrifuged at 500 × g for 5 minutes at 4 °C to obtain alveolar cells^44^. Cell suspensions were depleted of neutrophils, T-cells, and B-cells by immunomagnetic cell sorting (MACS) negative selection with anti-Ly6G, anti-CD3, and anti-CD19 (Biolegend and Miltenyi Biotec); Macrophages were isolated by MACS positive selection with anti-F4/80 (Miltenyi Biotec). Thus, total macrophages were obtained as the F4/80-positive, Ly6G/CD3/CD19-negative fraction. Subsequently, 1 mL of macrophage pellet (2 × 10^6^ cells/mL) was washed in FACS buffer stained with anti-CD11c-PE (BD Biosciences, USA) and anti-CD206-Alexa488 (BD Biosciences, USA) monoclonal antibodies and incubated on ice for 1-hour. After wells were washed in FACS buffer, they were re-suspended in 500 μL of FACS. Then, the numbers of M1 macrophage [F4/80(+)/CD11c(+)] or M2 macrophages [F4/80(+)/CD206(+)] per 1 mL of collected BAL fluid from rats of different groups were calculated in three independent experiments.

### Evaluation of the pulmonary relative MVD with IF study

The pulmonary angiogenesis was evaluated as relative MVD using CD31-FITC antibody to identify highest MVD area in fresh right lung of each rat^5^. Pulmonary vessels with a diameter of >100 μm were excluded from the analysis. Then, counting for CD31 (+) tubular structures was performed in the 5 highest MVD areas using ImageJ software (National Institutes of Health) and results were averaged. Meanwhile, pulmonary TNFα/total nitric oxide (NOx) levels and hepatic hydroxyproline measurement was measured by the Griess reaction and commercial available kits.

### Various measurements in gastrocnemius

By MACS positive selection, infiltrated macrophages were isolated from enzymatically digested right gastrocnemius as the F4/80-biotin-positive, Ly6G/CD3/CD19-negative fraction for analysis the portion (%) of M1 macrophages among total macrophages^21^. Briefly, the numbers of M1 [F4/80(+)/CD11c(+)] and M2 [F4/80(+)/CD206(+)] macrophages in 1 mL of muscle homogenates were calculated. In left gastrocnemius homogenates, the degree of muscular macrophage infiltration and cross section area of muscle fibers were evaluated by IHC staining with CD68 and α-sarcometric actin (myocyte cytoplasma) antibodies. Number of CD68(+) cells and cross section area in 10 microscope fields (x200) per muscle section were counted and expressed as cell/mm^2^ and μm. Three different sections in each tissue block were examined in each rat.

### Assessment of various *mRNA* and protein content/expressions

In bilateral anterior tibialis homogenates of rats of different groups, the protein content and 3-nitrotyrosine (marker of peroxynitrite) levels were measured in three independent experiments with BCA Protein Assay Kits (EMD Chemical, Darmstadt, Germany) and ELISA kits (Abcam) (Cambridge, MA, UK).

For each rat, total *RNA* and protein were extracted from bilateral anterior tibialis and left lung tissues for measurement of MCP-1, CD68, TNFα, NFkB-p65, iNOS, eNOS, ET-1, ET_B_R, CD31, VEGF, VEGFR2, p-VEGFR2, MyoD, MyoD1, MHC, MHC II, MuRF-1, MAFbX, p38MAPK, IL-4, IL-13 and β-actin expressions using appropriate antibodies and primers (Supplement Table 1) with SYBR green rt-qPCR and western blot analysis.

### *In vitro* studies with HPAEC and C2C12 myoblasts

The capacity of various agents on formation of capillary like tube structure and migration of HPAECs, between passages 2 and 4, were examined with Matrigel^®^ angiogenesis (Kurabo, Tokyo, Japan) and transwell chemotaxis filter migration assays^27^ in six independent experiments. For assessment of angiogenesis, matrigel was used to coat the wells of 24-well plates (0.25 ml per well) and was left to polymerize at 37 °C for 1 hour. After polymerization, the HPAECs (3 × 10^5^ cells) were incubated in growth media and allowed to attach for 24 hours in each well. Cells were washed twice with M199 and incubated for 6 hour with M199 containing 1% fetal calf serum and antibiotics.

According to the measured plasma TNFα (25–30 ng/mL) of BDL-V rats (Table 1), effects of incremental concentration of TNFα (10, 20 and 30 ng/mL) on the angiogenic and migration indices of HPAECs were evaluated in preliminary experiments (n = 3). Interestingly, the most potent stimulation of angiogenesis and migration were observed by 20 ng/mL of TNFα. Then, 20 ng/mL TNFα was used in the following *in vitro* experiments to combine with other test agents.

Overall, the HPAECs were fixed after 36-hour of different combined agents [buffer, TNFα (20 ng/mL); TNFα+thalidomide (thal, 10^−3^M) and AMG + TNFα + thalidomide with 2-hour AMG (100 μM, inhibitor of the iNOS) pre-treatment)]. Images were captured using an Olympus Inverted Research Microscope (Olympus, Tokyo, Japan) coupled to an Olympus C-5050 Zoom digital camera. Images were prepared in Adobe Photoshop 7.0 (Adobe, San Jose, CA, USA) and exported to an image analysis software package for identification of endothelial cell tubule-like networks. In four randomly chosen fields of each well, the angiogenic index was calculated by the ratio of total tubule length (≥30 μm) to the total area of the culture surfaces covered by HPAECs in the same fields. The angiogenic indices were obtained for each HPAECs culture well and compared among different treatment groups.

Meanwhile, HPAECs migration was assessed using a chemotaxis chamber with inserts equipped with a 8-mm-pore membrane of 0.3 cm^2^ and were placed in 24-well culture dishes, forming the upper and lower compartments of the assay, respectively (Transwell, Corning Costar, Cambridge, MA, USA). The lower compartments of the Boyden chamber, contained medium (buffer) alone (DMEM/0.2% BSA) or with different combined agents [buffer, TNFα, AMG, AMG+TNFα (with 2-hour AMG pre-treatment), TNFα+thalidomide and AMG + TNFα + thalidomide (with 2-hour AMG pre-treatment)], were pre-coated with type I collagen (50 mg/L). The upper compartment of the chemotaxis filter assay are seeded with HPAECs (3 × 10^5^ cells/mL), which had been serum-starved for 24 hours, in 150 μL of serum-free medium. The entire culture dish was incubated at 37 °C for 36-hour to allow possible migration of HPAECs. At the end of experiments, the non-migrated HPAECs remaining on the upper surface of the insert were removed with cotton tips. Inserts were washed three times with PBS, fixed in 100% methanol stained with May-Grunwald-Giemsa, mounted in glycergel on glass slides, and examined under microscope. HUVECs adhering to the underside of inserts were counted in 10 random high-power fields (×100) and expressed as percentages of the control (buffer only group). Then, the MI was calculated [MI = transmigrated HPAECs numbers in the presence of TNFα, AMG, AMG + TNFα, TNFα + thalidomide and AMG+TNFα+thalidomide, divided by the transmigrated HPAECs numbers in the absence of them (buffer only)] and compared among groups.

C2C12 mouse myoblasts were grown and near confluence cells were induced to differentiate by switching from a confluent DMEM media containing 20% fetal bovine serum to a differentiation DMEM media containing, 2% horse serum, penicillin/streptomycin antibiotics, and 50 mM HEPES, pH 7.4. Meanwhile, different combined agents [buffer, TNFα, AMG, AMG+TNFα (with 2-hour AMG pre-treatment), TNFα + thalidomide and AMG + TNFα + thalidomide] were added into differentiation media of C2C12 (3 × 10^5^) for 3 days. Then, myotube formation were assessed by immunofluorescence with anti-MyoD1 (nuclear)/anti-MHC (fiber) antibody, and visualized with AF488/FITC-conjugated secondary antibodies. A muscle cell containing 3 or more nuclei was considered as a myotube, as defined previously^22^,^23^. Total cell nuclei and nuclei within myotubes were counted using the NIH ImageJ software. Fusion index for day 3 myotubes was calculated as the number of MyoD1(+) nuclei in MHC(+) myotubes (cells containing 3 or more nuclei) to the total number of nuclei in one field for five random microscopic fields. To analyze day 3 myotube diameter, five fields were chosen randomly, and three myotubes were measured per field along the long axis. Then, fusion indices and myotube diameters were compared among different treatment groups.

### Preparation primary rat CD16^+^ monocytes for *in vitro* co-culture studies

Among the second set of animals, after BW/MAP measurement and mid-abdominal incision for liver and spleen size gross inspection, the heparinized whole body blood (25–30 mL from each rat) collected from whole body under anesthesia was divided into two parts after. The first part, 10 mL of heparinized blood collected from each rat, was used for TNFα, MCP-1, sICAM-1, VEGF, alanine various serologic markers measurements. The second parts, 15 mL of heparinized blood from each rat, was pooled [total 75 mL of whole blood from each group of BDL-V, BDL-thal, C-V, C-thal rats (n = 6 in each group)] to prepare CD16+ monocyte fractions using above description protocol for co-culture with either HPAECs or C2C12 myoblasts. Approximately, 25–30 × 10^6^ CD16^+^ monocytes was obtained from each group of BDL-V, BDL-thal, S-V or S-thal rats.

### *In vitro* effects of co-culture of CD16^+^ monocytes with HPAECs/C2C12 myoblasts

The capacity of CD16^+^ monocytes collected from S-V, S-thal, BDL-V and BDL-thal rats to modulate angiogenic and migration index of cultured HPAECs were examined in six independent experiments. Briefly, HPAECs (3 × 10^5^) were cultured in duplicate with and without equal amount of CD16^+^ monocytes that re-suspended in RPMI 1640 containing 10% FCS and antibiotics. As a control, CD16^+^ monocytes were also cultured alone on Matrigel^®^ and transwell chemotaxis assay systems. After 36 hours, the total tube lengths and transmigrating cells in each well were measured in different co-cultured HPAECs + CD16^+^ monocytes systems. Then, the relative angiogenic and migration indices were calculated by the ratio of these two parameters in the co-cultured HPAECs + CD16^+^ monocytes system to those in the mono-cultured HPAECs system was calculated. Additionally, supernatants of co-cultured HPAECs + different CD16^+^ monocytes were collected as conditioned medium, and used for the second Matrigel^®^ angiogenesis and transwell chemotaxis filter migration assays in mono-cultured HPAECs system. Again, the relative angiogenic and migration indices were calculated by the ratio of these two parameters in the HPAECs system that incubated with supernatant of co-cultured HPAECs + different CD16^+^ monocytes to those without co-incubation with above-mentioned supernatant (mono-cultured HPAECs).

Similarly, the capacity of CD16^+^ monocytes (3 × 10^5^) collected from different rats to modulate fusion index and myotube diameter of mono-cultured C2C12 cells (3 × 10^5^) were examined. Briefly, the relative fusion index and myotube diameter were calculated by the ratio of these two parameters in the co-cultured C2C12 + different CD16^+^ monocytes system to those in the mono-cultured C2C12 cells system was calculated. Additionally, supernatants of co-cultured C2C12 + different CD16^+^ monocytes were collected as conditioned medium, and used for the second myotubes formation assays in C2C12 cells system. Finally, the relative fusion index and myotube diameter were calculated by the ratio of these two parameters in the C2C12 system that incubated with supernatant of co-cultured C2C12 + different CD16^+^ monocytes to those without co-incubation with above-mentioned supernatant (mono-cultured C2C12s).

### Cell lysates for various mRNA measurements

Cultured HPAECs or day 3 C2C12 myoblasts (3 × 10^5^) with 36-hour of buffer, TNFα, TNFα+thalidomide and AMG + TNFα + thalidomide pre-treatment as well as co-cultured HPAEC + different CD16^+^ monocytes or co-cultured C2C12 + different CD16^+^ monocytes were lysed for obtaining cell lysates and subsequently total RNAs extraction. Then, various *mRNA* expressions were measured in six independent experiments.

### Statistical analysis

Data were expressed as means ± S.D. Statistical significance in each group was determined using one-way ANOVA with post hoc multiple comparisons or student’s *t* test. When criteria for parametric testing were violated, the Mann-Whitney U-test or Kruskal-Wallis test was performed. Significance was determined at a p-value less than 0.05.

## Additional Information

**How to cite this article**: Li, T.-H. *et al*. Down-regulation of common NFκB-iNOS pathway by chronic Thalidomide treatment improves Hepatopulmonary Syndrome and Muscle Wasting in rats with Biliary Cirrhosis. *Sci. Rep.* **6**, 39405; doi: 10.1038/srep39405 (2016).

**Publisher's note:** Springer Nature remains neutral with regard to jurisdictional claims in published maps and institutional affiliations.

## Supplementary Material

Supplementary Information

## Figures and Tables

**Table 1:** Basal characteristics of all rats (n = 7 in each group).

	S-V	S-thal	BDL-V	BDL-thal
Mean arterial pressure (MAP, mmHg)	116.2 ± 6.5	110.9 ± 8.1	99.4 ± 6.7^#^	100.3 ± 9.8
Heart rates (HR, /min)	390 ± 12	408 ± 310	419 ± 18	431 ± 19
Plasma TNFα level (pg/mL)	306 ± 29	359 ± 63	2803 ± 361^##^	1871 ± 240**
Plasma MCP-1 level (ng/mL)	65 ± 18	58 ± 13	390 ± 98^#^	381 ± 79
Plasma sICAM-1 level (ng/mL)	28.2 ± 8.5	23.5 ± 11.2	37.1 ± 6.5^#^	30.4 ± 5.8*
Plasma VEGF level (pg/mL)	43 ± 5	32 ± 6	69 ± 5^#^	61 ± 8
Plasma ALT (U/L) level	48 ± 5	54 ± 2	191 ± 8.2^#^	87.5 ± 12.1*
Plasma AST (U/L) level	56 ± 2	69 ± 4	350 ± 21^##^	209 ± 8.2**
Percentage (%) of circulating CD16^+^ (inflammatory) monocyte in total monocytes	31.3 ± 1.5	30.4 ± 2.6	47.8 ± 6.8^#^	36.9 ± 1.5*
Hepatic hydroxyproline content (μg/g)	242 ± 45	179 ± 41	608 ± 55^##^	465 ± 60*

^#,##^*p* < 0.05, 0.01 *vs*. S-V; *^,^**p < 0.05, 0.01 *vs*. BDL-V.

**Table 2:** Muscle wasting parameters (n = 7 in each group).

	S-V	S-thal	BDL-V	BDL-thal
Body wt (g)	426 ± 10.8	410 ± 9.9	419 ± 11.3	399 ± 11.7
Absolute anterior tibialis wet wt (g)	1.43 ± 0.12	1.3 ± 0.21	0.72 ± 0.07^#^	0.99 ± 0.51
Relative anterior tibialis wet wt/body wt (mg/g,%)	0.0035 ± 0.0009	0.0032 ± 0.0004	0.0017 ± 0.0006^#^	0.0025 ± 0.0005*
Absolute gastrocnemius wet wt (g)	4.4 ± 0.006	4.19 ± 0.031	3.11 ± 0.03^#^	3.89 ± 0.081*
Relative gastrocnemius wet wt/body wt (mg/g,%)	0.0103 ± 0.0009	0.0102 ± 0.0002	0.0073 ± 0.0004^#^	0.0097 ± 0.0012*
Protein content (mg/g)	246 ± 21	239 ± 38	151 ± 9^#^	211 ± 10*

^#,##^*p* < 0.05, 0.01 *vs*. S-V; ^*,**^p < 0.05, 0.01 *vs*. BDL-V. Wt: weight.
