# Supplementary material for: Down-regulation of common NFκB-iNOS pathway by chronic Thalidomide treatment improves Hepatopulmonary Syndrome and Muscle Wasting in rats with Biliary Cirrhosis
Source: Sci Rep. 2016 Dec 23;6:39405. doi: 10.1038/srep39405 (PMC5180197; doi:10.1038/srep39405)
Supplement: Supplementary Information [file srep39405-s1.doc]

**Down-regulation of common NFκB-iNOS pathway by chronic Thalidomide treatment improves Hepatopulmonary Syndrome and Muscle Wasting in rats with Biliary Cirrhosis**

Tzu-Hao Li, Pei-Chang Lee, Kuei-Chuan Lee, Yun-Cheng Hsieh, Chang-Youh Tsai, Ying-Ying Yang, Shiang-Fen Huang, Tung-Hu Tsai, Shie-Liang Hsieh, Ming-Chieh Hou, Han-Chieh Lin & Shou-Dong Lee

| **Gene name** | **Primers sequences** |
| --- | --- |
| TNFα | For:5′-gctcacaatgtctgtgcttagag-3′; Rev:5′-gcagtagccacagctccag-3′ |
| NFkB-p65 | For:5′- cacctcaatggctacacacaggacca-3′; Rev:5′-atcttgagctcggcagtgtt-3′ |
| iNOS | For:5′-agcatcacccctgtgttccaccc-3′; Rev:5′-tggggcagtctccattgcca-3′. |
| ET-1 | For:5’-gctcctgctcctccttgatg-3’; Rev:5’-ctcgctctatgtaagtcatgg-3’ |
| ET_B_R | For:5’-agctgggtgcccttcatacagaaggc-3’;Rev:5’-tgcacacctttccgcaagcacg-3’ |
| CD68 | For:5′-gctacatggcggtggagtacaa-3′; Rev:5′-atgatgagaggcagcaagatgg-3′ |
| MCP-1 | For:5′-atgcagttaatgccccactc-3′;Rev:5′-tgctgctggtgattgtcttg-3′ |
| ICAM-1 | For:5′-cctgtttcctgcctctgaag-3′;Rev:5′-gtctgctgagacccctcttg-3′ |
| CD31 | For:5′-cttcaccatccagaaggaagagac-3′; Rev:5′-cactggtattccatgtctctggtg-3′ |
| VEGF | For:5′-ctacctccaccatgccaagt-3′; Rev:5′-gcagtagctgcgctgataga-3′ |
| VEGFR2 | For:5'-aagcaaatgctcagcaggat-3'; Rev:5'-taggcagggagagtccagaa-3' |
| Caspase-3 | For:5'-tgactggaaagccgaaactc-3';Rev:5'-agcctccaccggtatcttct-3′. |
| Caspase-8 | For:5'-ccgagctggacttgtgacc-3';Rev:5'-ctgcccagttcttcagcaat-3′. |
| ROCK-1 | For:5'- acctgtaacccaaggagatgtg-3';Rev:5'-cacaattggcaggaaagtgg-3′. |
| ROCK-2 | For:5'-aagtgggttagtaggttg-3';Rev:5'-ggcagttagctaggtttg-3′. |
| p38MAPKα | For:5'-cgaaatgaccggctacgtgg-3';Rev:cacttcatcgtaggtcaggc-3′. |
| MAFbx | For:5'-agaaaagcggcaccttcgt-3';Rev:cttggctgcaacatcgtagtt-3′ |
| MyoD | For:5′-ccgcctgagcaaagtaaatga-3′;Rev:5′-gcaaccgctggtttggatt-3′. |
| MHC II | For: 5′-aaggtcggcaatgagtatgtca-3′;Rev:5′- caaccatccacaggacactcttc-3′. |
| IL-4 | For:5′-ggatgtgccaaacgtcctc-3′;Rev:5′-gagttcttcttcaagcatggag-3′. |
| IL-13 | For:5′-ctttctttagcggccac-3′;Rev:5′-cagagcgccatgaagcccagag-3′ |
| 18S | For:5′-gtaacccgttgaaccccatt-3′; Rev:5′-ccatccaatcggtagtagcg-3 |

**Supplement Table 1. Primer of rat gene used for quantitative realtime PCR analysis.** TNFα: Tumor necrosis factor-alpha; NFκB:Nuclear factor kappa B, iNOS, inducible nitric oxide synthase; MCP-1:monocyte chemoattractant protein-1; ICAM-1: intercellular adhesion molecule-1; VEGF: Vascular endothelial growth factor; p38MAPK: p38 mitogen activated protein; MAFbx: Muscle atrophy F-box; MyoD: Myogenic differentiation; MHC: Myosin heavy chain; IL-4: Interleukin-4; IL-13: Interleukin-13

| **Fold changes from 18S** | **S-V** | **S-thal** | **BDL-V** | **BDL-thal** |
| --- | --- | --- | --- | --- |
| **ET-1(/18S)** | 0.85±0.087 | 0.87±0.021 | 1.449±0.052^##^ | 1.38±0.094 |
| **ET_B_R(/18S)** | 0.77±0.081 | 0.76±0.0930 | 1.62±0.032^#^ | 1. 60±0.0819 |
| **eNOS(/18S)** | 0.920±0.0511 | 0.84±0.0121 | 1.13±0.0597^##^ | 1.15±0.0867 |

**Supplement Table 2. *mRNA* expression of genes on the ET-1-ETB-eNOS pathway in lung tissue between groups (n=7).** ET-1: Endothelin-1; ET_B_R: Endothelin B receptor; ^#,##^*p*<0.05, 0.01 *vs*. S-V

|  | **Conentration of acute TNFα incubation in HPAECs** | | | |
| --- | --- | --- | --- | --- |
| **Fold changes from 18S** | **Buffer only** | **10ng/mL TNFα** | **20ng/mL TNFα** | **30ng/mL TNFα** |
| **caspase-3 (/18S)** | 0.348±0.015 | 0.523±0.012 | 1.61±0.48^#^ | 1.94±0.13 |
| **caspase-8 (/18S)** | 0.206±0.029 | 0.638±0.047 | 0.957±0.087 | 0.913±0.104 |
| **ROCK 1 (/18S)** | 0.352±0.041 | 0.71±0.082 | 1.84±0.16^##^ | 2.03±0.013 |
| **ROCK 2 (/18S)** | 0.243±0.036 | 0.613±0.087 | 0.76±0.049 | 0.81±0.054 |

**Supplement Table 3. *mRNA* expression of endothelial cells migratory genes-caspase-3&8 ROCK 1&2 in cell lysates of HPAECs that incubated with incremental concentration of TNFα (n=6).** ROCK: Rho kinase; ^#,##^*p*<0.05, 0.01 *vs*. 10ng/mL TNFα-incubated group

**Suppl. Fig. 1 (A-G): Original blots for some cropped and high-contrast protein bands in Fig. 2B and 4C.**

**
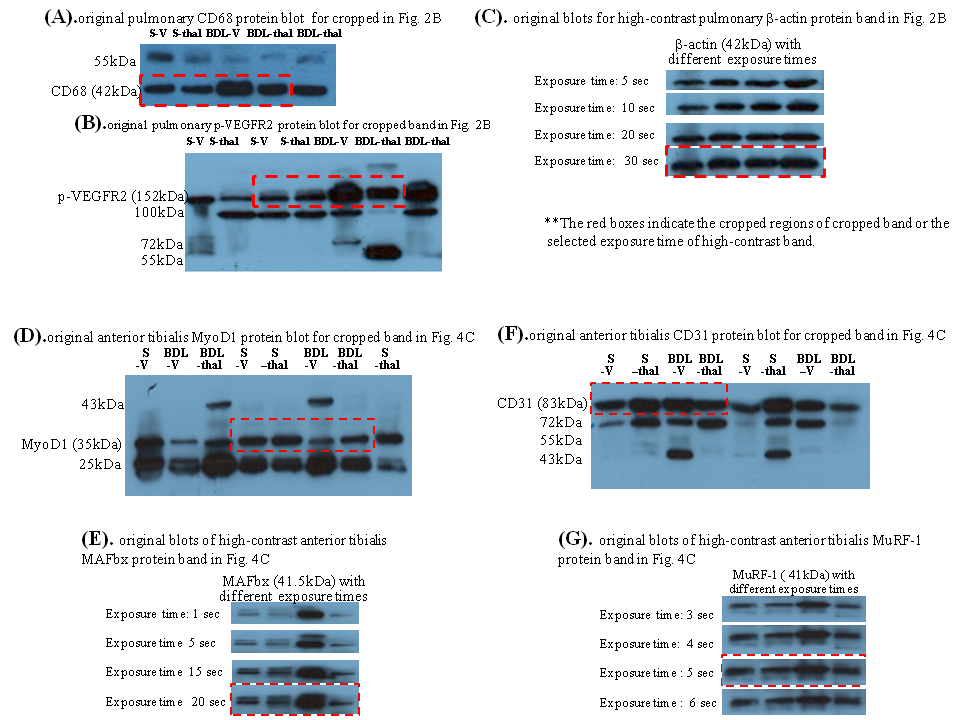
**

**Supplement materials and Methods**

***Evaluation of the pulmonary vascular density with IF study.*** The pulmonary angiogenesis (pulmonary window vascular length and area) were measured with flow probes and FITC-labeled CD31 IF staining. Briefly, 4 to 5 vascularized pulmonary windows (wedge-shaped regions of connective tissue bordered by the alveolar wall and the its blood vessel pairs) of right lung were dissected free, washed in phosphate-buffered saline, dried on gelatin slides, and fixed in 100% MeOH (-20 ℃ for 30 min). Slides were then incubated overnight at 4°C with the primary antibody mouse anti-rat CD31-biotin [1:200; AbD Serotec, Oxford, UK]. Then secondary antibody [CY2-conjugated streptavidin, 1:1000; Jackson ImmunoResearch, West Grove, PA, USA] was applied for 1 hour at room temperature**.** For each pulmonary window, at least four sets of (100x)-magnification immunofluorecent images were assessed using a upright fluorescent microscope (AX80, Olympus, Japan) with charge-couple device (QICAM, High-performance IEEE 1394 FireWireTM Digital CCD Camera, Q IMAGING, BD, Canada) and threshold by Image J software (available for download from the National Institutes of Health (NIH, http://rsb.info.nih.gov/ij/). The vascular length was manually measured with the pencil tool and the vascular area automatically with histogram function, respectively. According to the information provided by QICAM, with the eyepiece 10x, the diameter of one pixel on an image taken with QICAM equals to 4.65 μm. Under 100x-magnification (objective 10x and eyepiece 10x), the diameter of one pixel would be 4.65 μm/10=0.465 μm. The vascular length could thus be determined accordingly. The unit of vascular length per unit area of pulmonary window would be μm·(μm^2^)^-1^= μm^-1^ and the vascular area per unit area of pulmonary window, actually, could be pixel·pixel^-1^ without being converted toμm^2^·(μm^2^)^-1^

#### ***Muscle macrophage isolation by MACS.*** Right gastrocnemius was enzymatically digested; cell suspensions were depleted of neutrophils, T-cells, and B-cells by immuno-magnetic sorting beads through MACS negative selection with Ly6G-biotin, CD3-biotin, and CD19-biotin antibodies. By MACS positive selection, macrophages were isolated from above mention cell suspension with as the F4/80-biotin-positive, Ly6G/CD3/CD19-negative fraction.

Then, the cell pellet was washed in FACS buffer for staining with F4/80-FITC, CD11c-PE and CD206-AF488 antibodies and incubated on ice for 1-hour. After well were washed in FACS buffer, they were re-suspended in 500μL of FACS and analyzed with FACS Calibur flowcytometer. Then, the number of M1[F4/80(+)/CD11c(+)] and M2[F4/80(+)/CD206(+)] macrophages in 1mL of muscle homogenates was calculated.

***Muscular IHC studies.*** The left gastrocnemius was fixed by immersion in 10% phosphate-buffered formalin. Then, degree of muscular macrophage infiltration and cross section area of muscle fibers were evaluated by IHC staining with CD68 and α-sarcomeric actin (myocyte cytoplasma) antibodies in 10 microscope fields from three different sections in each tissue block, and calculated with Image Pro Plus software (Media Cybernetics, Bethesda, MD, USA) on the images with the dimensions of 900×900 µm.

***Skeletal muslce protein content and 3-nitrotyrosine measurement.*** The protein content of the anterior tibilais homogenates was measured using BCA Protein Assay Kits (EMD Chemical, Darmstadt, Germany). In brief, 12.5μL of homogenate was added to duplicate wells of 96-well plates followed by 200 μL of BCA solution. The absorbance was measured at 562 nm and compared with a standard curve. Then, the 3-nitrotyrosine, as marker of peroxynitrite formation, levels in the anterior tibilais tissue homogenates was measured with ELISA kits (Abcam) (Cambridge, MA, UK).

**Cell lysates for various mRNA measurements.** Cultured HPAECs or day 3 C2C12 myoblasts (3×10^5^) with 36-hour of buffer, TNFα, TNFα+thalidomide and AMG+TNFα+thalidomide pre-treatment were washed twice with ice-cold phosphate-buffered saline. Cells were lysed using RIPA buffer [20mM MOPS, 150mM NaCl, 1mM EDTA, 1% Igepal, 1% sodium deoxycholate and 0.1% SDS supplement with 1:1000 concentration of protease inhibitor mix (Sigma)]. Similarly, the cell lysates of co-cultured HPAEC+different CD16^+^ monocytes or co-cultured C2C12+different CD16^+^ monocytes were obtained. The lysates were centrifuges at 10,400×g for 10 minutes at 4°C and kept at -80°C until use. Total RNAs were obtained from cell lysates using RNeasy extraction kit (Qiagen, Mississauga, ON, Canada). cDNA was synthesized from 1μg total RNA using the QuantiTect Reverse Transcription kit (Qiagen). Then, various *mRNA* expressions [NFkB-p65, iNOS, VEGF, VEGFR2 for HPAECs or HPAECs+different CD16^+^ monocytes; NFκBp65, iNOS, MAFbx, MuRF-1, MyoD and MHCII for C2C12 myoblasts or C2C12s+ different CD16^+^ monocytes] were measured in six independent experiments.

**Materials.** All the antibodies [CD16-PE, CD14-FITC, TNFα-APC, NFκBp65-APC, MCP-1-APC, iNOS-APC, Ly6G-biotin, CD3-biotin, CD19-biotin, F4/80-biotin, F4/80-FITC, CD11c-PE, CD206-AF488, CD31-FITC, secondary F488/FITC–conjugated, CD65, CD68, α-sarcomeric actin, NFkB-p65, MyoD1, MHC, MuRF-1, MAFbx, VEGF, p-VEGFR2, and β-actin] for flow cytometry, IHC, IF and western blot were purchased from BD Biosciences; R&D system (Minneapolis, MN); Santa Cruz (Biotechnology, Inc.); Sigma-Aldrich (St. Louis, MO, USA) and Abcam (Cambridge, MA, UK). Negative selection immuno-magnetic beads and MACS isolation kits were purchased from Miltenyi Biotec (Bergisch Gladbach, Germany). TNFα, VEGF, MCP-1, sICAM-1, NOx and 3-nitrotyrosine ELISA/colorimetric assay kits were purchased from R&D system (Minneapolis, MN); BMS250, Bender MedSystems (GmbH Vienna, Austria); Biovision; eBioscience; Cayman Chemicals (MI, USA) and Abcam. HPAECs were purchased from Lonza Clonetics (Walkersville, MD, USA). C2C12 myoblasts were purchased from Bioresource Collection and Research Center (Taiwan). Other unlisted materials were purchased from Invitrogen and Sigma-Aldrich (St. Louis, MO, USA).
